# Use of ^1^H NMR to Detect the Percentage of Pure Fruit Juices in Blends

**DOI:** 10.3390/molecules24142592

**Published:** 2019-07-17

**Authors:** Lucia Marchetti, Federica Pellati, Stefania Benvenuti, Davide Bertelli

**Affiliations:** 1 Department of Life Sciences, University of Modena and Reggio Emilia, Via G. Campi 103, 41125 Modena, Italy; 2 Doctorate School in Clinical and Experimental Medicine (CEM), University of Modena and Reggio Emilia, 41125 Modena, Italy

**Keywords:** fruit juice, blends, adulteration, ^1^H NMR, PLS, chemometrics

## Abstract

The consumption of high-nutritional-value juice blends is increasing worldwide and, considering the large market volume, fraud and adulteration represent an ongoing problem. Therefore, advanced anti-fraud tools are needed. This study aims to verify the potential of ^1^H NMR combined with partial least squares regression (PLS) to determine the relative percentage of pure fruit juices in commercial blends. Apple, orange, pineapple, and pomegranate juices were selected to set up an experimental plan and then mixed in different proportions according to a central composite design (CCD). NOESY (nuclear Overhauser enhancement spectroscopy) experiments that suppress the water signal were used. Considering the high complexity of the spectra, it was necessary to pretreat and then analyze by chemometric tools the large amount of information contained in the raw data. PLS analysis was performed using venetian-blind internal cross-validation, and the model was established using different chemometric indicators (RMSEC, RMSECV, RMSEP, R^2^_CAL_, R^2^_CV_, R^2^_PRED_). PLS produced the best model, using five factors explaining 94.51 and 88.62% of the total variance in X and Y, respectively. The present work shows the feasibility and advantages of using ^1^H NMR spectral data in combination with multivariate analysis to develop and optimize calibration models potentially useful for detecting fruit juice adulteration.

## 1. Introduction

Fruit juice consumption is increasing worldwide since fruit-based products are promoted as healthy foods and, in addition, producers try to be more and more innovative by developing the market segment of blends and mixed juices of high nutritional value. According to the European Fruit Juice Association (AIJN), consumption of 38.5 million L of fruit juice was registered worldwide in 2015 and, in the same year, the consumption estimated per head of population was about 19 L in the European Union, 26 L in North America, 6 L in South America and 2 L in Pacific Asia [1]. Considering the large market volume, economic frauds and adulteration in this sector represent an ongoing problem and have often been reported; hence, there is a need for advanced and suitable anti-fraud tools [2,3]. Tackling the total cost of analysis to assess fruit juice authenticity can be very expensive; mostly the aim is to detect water dilution, the addition of inexpensive juice blends to higher-value fruit juice, or the addition of pure beet sugar. Currently, the most common techniques applied to reveal fraud in this field involve whole-food profiling or the search for a number of compounds (targeted analysis). The main disadvantages of these analyses are the high cost and time-consuming nature, the fact that adulterants at concentrations lower than 10% are difficult to detect, and the fact that, in most cases, only one type of adulteration can be unmasked at a time [4]. 

For this reason, advanced analytical methods are highly recommended to prevent fraudulent practices and to protect the rights of producers as well as those of consumers, with particular attention to safety issues. Since many factors contribute to the variation in juice composition, e.g., fruit geographical origin and climate, maturity degree, technological processes, and storage conditions, a comprehensive method based on the fingerprinting approach is recommended, in which a large number of unknown metabolites is included in the analysis and contributes to the results. For this purpose, the possibility of using nuclear magnetic resonance (NMR) spectroscopy for monitoring fruit juice and other fruit-derived products is well known and has been demonstrated to be an efficient tool in beverage authentication, since the spectral data cover a wide range of compounds [5]. NMR has many advantages over the most common separation methods based on GC and HPLC, which may be cheaper but they seem to be more appropriate for target analysis [6]. Indeed, NMR can detect many different compounds in one sample run, it is non-destructive, stable over time, and it requires only a limited sample preparation [6]. Various applications of NMR spectroscopy are now available for beverage quality control, as reported for wines, spirits, and juices [7,8]. ^1^H NMR spectroscopy has shown great potential for determining the country of origin of green tea samples [9]. Moreover, the same technique has been demonstrated to be very accurate in the determination of the origin of fruit, and it can be used to examine the source of the raw material used in the preparation of juices [10]. NMR spectroscopy has also been recently applied to alcoholic beverages for authentication purposes, taking into account their high prices and the high risk of fraud by adulteration or deliberate mislabeling. The potential of NMR spectroscopy has also been evaluated to verify the composition of beer and to correlate it to the brewing site and to the date of production, as well as for quality control [11]. In particular, the use of NMR spectroscopy has become an indispensable tool for authenticity studies on fruit juice and has now reached the commercial level. The NMR technique is able to evaluate simultaneously, from a single dataset, a multitude of parameters related to the quality and authenticity of juice, providing targeted and non-targeted multi-marker analysis. In addition, the spectral results can be compared with databases of reference juice [10,12,13]. However, to the best of our knowledge, NMR application for the qualitative and quantitative determination of the composition in juice blends has not been previously described in the literature.

The application of ^1^H NMR to complex matrices, as in the case of fruit juice, which normally contains a large amount of natural or added sugars and organic acids, and lower but significant amounts of other organic substances (phenolics, terpenes, amino acids, etc.), may result in complex spectra, with a lot of crowded signals. This condition often makes it difficult to define the assignments of substances and to correctly proceed with the integration without the use of deconvolution. Deconvolution is an algorithm-based process that allows us to resolve or decompose a set of overlapping peaks into their separate additive components. In these cases, a non-targeted approach based on a sample fingerprint is advantageous. In particular, the use of the entire ^1^H NMR spectrum profile without any kind of targeted measurement was evaluated in this work. Fingerprinting techniques require the use of adequate statistical methods to extract information from raw data. Chemometrics can reveal latent correlations in the data and can be useful for both qualitative and quantitative purposes. 

Partial least squares (PLS) modeling is a powerful multivariate statistical tool that has often been applied to spectral analysis. PLS is related to other multivariate calibration methods, such as classical least squares (CLS), inverse least squares (ILS), and principal component regression (PCR) methods [14]. The main scope of PLS is to eliminate multicollinearity in the set of explanatory variables X of a regression model, reducing the dimension of the set in such a way that the resulting subset of descriptive variables is optimal for predicting the dependent variable Y. The values of Y typically represent the analyte concentrations (or any sample properties) [14]. For the quantitative approach, PLS is the most frequently used multivariate statistical method. Hence, this study aims to verify the potential of proton NMR (^1^H NMR) combined with PLS to construct models for the determination of the relative percentage of pure fruit juices in a blend.

## 2. Results and Discussion

The use of ^1^H NMR coupled with PLS was proposed here to develop and optimize multivariate calibration models to determine the relative concentration of four pure juices in blends. The PLS model was established by using different chemometric indicators (root mean square error of calibration (RMSEC); root mean square error of cross-validation (RMSECV); root mean square error of prediction (RMSEP); coefficient of determination for calibration (R^2^_CAL_), cross-validation (R^2^_CV_) and prediction (R^2^_PRED_).

**Figure 1:**
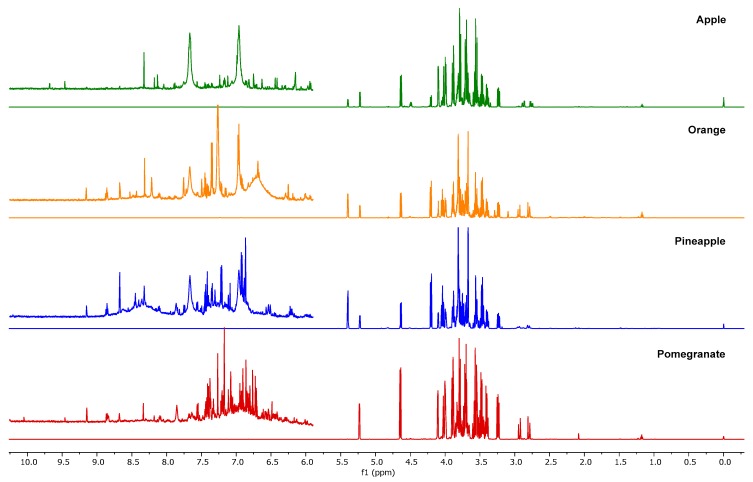
1D NOESY spectra of four pure juices, with the 6–10 ppm regions enlarged.

**Figure 2:**
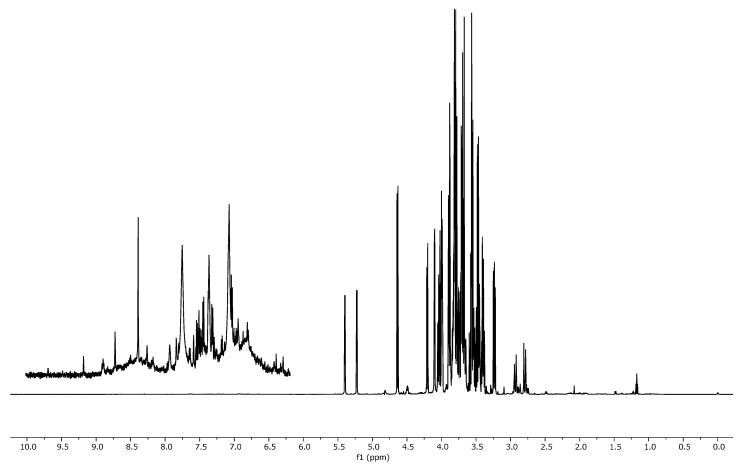
Typical 1D NOESY spectrum of mixture containing equal percentages of pure apple, orange, pineapple, and pomegranate juice.

Figure 1 shows the NMR spectra of the four pure juices considered, and Figure 2 shows the typical spectrum of a mixture containing equal percentages of apple, orange, pineapple, and pomegranate juice. As expected, the spectra present wide regions with overlapping phenomena, which makes it difficult to proceed with the peak integration. The typical ^1^H NMR spectrum of a juice shows three defined regions. In the first, ranging from 0.5 to 3.0 ppm, protons of organic acids (citric and malic) and amino acids (alanine, valine, and proline) are present. The second region (3.0–6.0 ppm) is typical for carbohydrates, with sucrose, α-glucose, β-glucose, and fructose being the most abundant [7]. The last region, ranging from 6.0 to 8.5 ppm, shows phenolic metabolites and aromatic protons. Moreover, each juice presents the peak assigned to the methyl group of ethanol at 1.17 ppm [6,15]. Spectra of pure juice have similar shapes in the aliphatic region and limited quantitative differences, except for the two peaks at 5.40 and 4.22 ppm related to sucrose, which is absent in pomegranate. The region ranging from 6 to 10 ppm is meanwhile more typical; here, aromatic and phenolic compounds are normally present. In this case, the signals are lower in intensity with respect to the aliphatics. As regards the mixtures, the enlargement of the aromatic region in Figure 2 appears even more complex, due to the fact that the weak aromatic signals of each single juice are more diluted in the blend and the number of signals is increased, owing to the simultaneous presence of many compounds. All these considerations led us to choose an untargeted approach combined with the consolidated PLS chemometric method [16].

The number of latent variables to be included in the PLS model was selected in such a way that the RMSEC, RMSECV (obtained from calibration and internal cross-validation, respectively) and RESEP (obtained from external validation test set) were reduced to the lowest values, ensuring at the same time the highest possible predictive capacity. Other statistical parameters to be considered are R^2^ and the amount of explained variance. The best model was built by using five factors, explaining 94.51% and 88.62% of total variance in X and Y, respectively. All the calculated RMSEs (except RMSECV for pineapple and pomegranate) are lower than 10 and each R^2^ value is acceptable, especially for prediction values. All the chemometric indicators are reported in Table 1.

**Figure 3:**
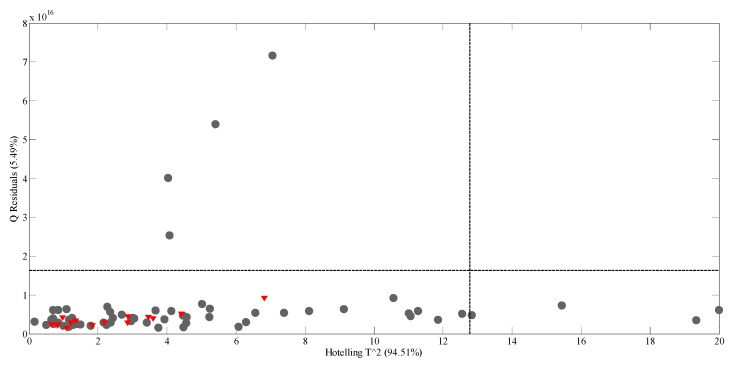
Q residuals versus Hotelling’s T^2^ plot for the PLS model of 60 fruit juice samples (•) and test set samples (▼).

In Figure 3 the Hotelling’s T^2^ vs. Q residuals graphic is shown. The presence of potential spectral outliers was verified by applying a 95% confidence interval, any outlier is present. Four samples with high Q residuals and three with high T^2^ scores are present, but none of them was excluded from the model. All the test set samples (shown in red) are in the 95% confidence area, demonstrating the high predictive capacity of the model. 

**Figure 4:**
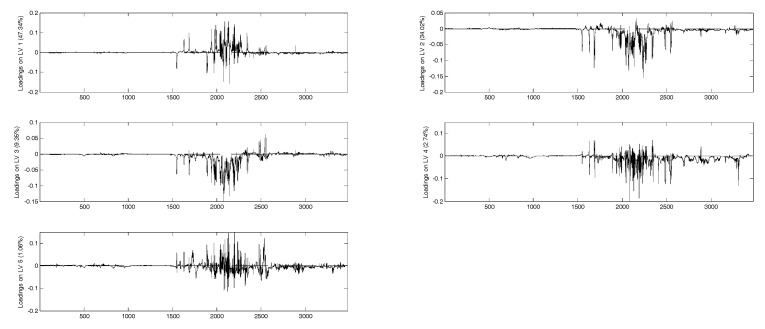
Loadings for the five extracted latent variables.

**Figure 5:**
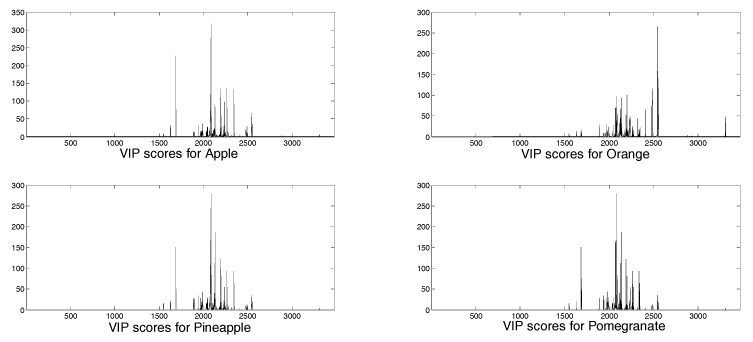
VIP scores obtained from the selected model for the four juices.

In Figure 4 and Figure 5 the loadings for the five extracted latent variables and the VIP (variable importance in the projection) scores, respectively, are shown for each juice. A VIP score is a measure of a variable importance in the model. It summarizes the contribution of a variable to the model. The VIP score is calculated as a weighted sum of the squared correlations between the PLS components and the original variables. As regards loadings, highly correlated variables have similar weights in the loading vectors. Thus, a different pattern of variables is significant for each extracted factor. In this case, for all the latent variables any significance arises for the aromatic signals, even though this spectral region (6–8 ppm) seems to be more characteristic for each juice in comparison with the aliphatic areas (3–4 ppm), which appear quite similar (Figure 1). Considering VIP scores, apple, pineapple, and pomegranate share the same pattern of variables, while in the case of orange juice the most important signals are those located at low frequencies, corresponding to acid compounds. In any case, and also for orange juice, the significance of the aromatic and phenolic region is limited. Some different pre-treatment procedures were attempted to increase the significance of the aromatic spectral region, but no substantial improvement was achieved.

**Figure 6:**
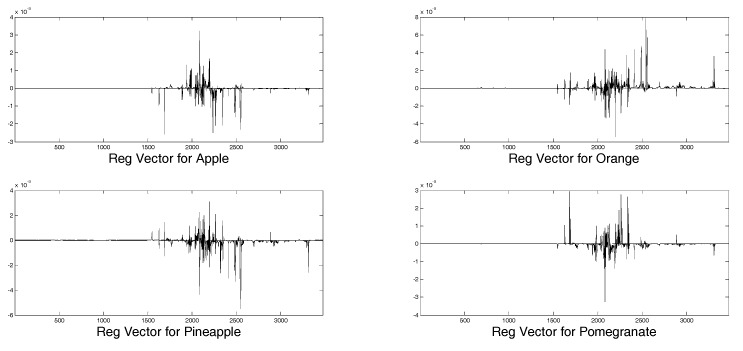
Regression vectors for the four juices considered in the model.

The regression vectors for each juice are shown in Figure 6. The four plots are different from each other, confirming the same tendency of VIP scores, thus in the definition of regression models different variables play a significant role for each juice. 

**Figure 7:**
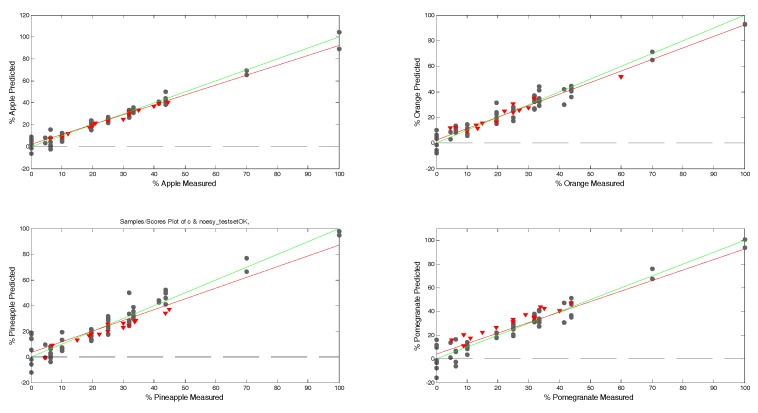
Correlation between the prediction and measured values of juice samples, showing calibration (•) and test set (▼) samples.

Figure 7 shows the scatter plot of measured values vs. predicted values in mixture samples for calibration and test sets (regression data are reported in Table 1). The regression models are similar in quality and predictive capacity. The best-performing model is apple, while the worst is pomegranate (Figure 7: top left and bottom right, respectively).

The method discussed here is suitable and sufficiently effective for the quantification of the considered mixtures. To validate this approach, the next step will be the evaluation of the predictive capacity of mixtures prepared with juices of other brands and also of mixtures in which one or more components are different from those covered by the model. Moreover, this method showed its effectiveness in a wide range of component percentages (6.25–100%), thus proving useful for both high and low concentrations. As a consequence, it should also be able to detect small differences from the composition declared on the labels of the commercial products.

The results presented herein show that the NMR analysis coupled with chemometrics provides adequate results in a comparable time with respect to other analytical approaches. The main advantages of the method proposed include the reduced sample preparation and lack of extraction and purification steps. In addition, this technique is effective at reducing the amounts of reagents and solvents, making it more competitive and environmentally sustainable than the most common separative techniques.

## 3. Materials and Methods

### 3.1. Samples

To verify the feasibility of ^1^H NMR combined with chemometrics to determine the percentage of pure fruit juices in a mixture, the following experimental plan was designed. To minimize the system complexity in this step of the research, four fruit juices (apple, orange, pineapple, and pomegranate) from a single brand were purchased from a local marketplace. These samples represent some of the most consumed fruit juices, often combined for the preparation of more attractive mixtures for consumers. 

To obtain the highest significant distribution of mixtures to be analyzed, they were prepared in accordance with an experimental plan, obtained by means of a central composite design (CCD), with α = 1.4826, based on a 2^4^ full factorial design, plus eight axial points, plus two replicates in the center of the domain and four pure juice samples [17]. The ratios provided by the model were transformed into a percentage composition to get the final experimental plan and then were used as dependent variables for the subsequent PLS analysis (Table 2). 

Analyses were performed in duplicate, making sure to repeat the analysis on different days. In this way, the 60 experiments were conducted in a randomized order. The intra- and inter-day variability, previously evaluated on one sample, were analyzed 10 times on the same and on different days, giving 1.4 and 4.0 CV%, respectively. In addition, an external validation test set was constructed including five randomly selected CCD replicates (newly prepared and analyzed) and 10 extra mixtures outside the CCD, with random percentages of the four juices (Table 3).

For the preparation of samples, each juice was first centrifuged at 5000 rpm for 5 min to remove all the material in suspension. Subsequently, for NMR analysis, different juices were mixed together in the ratios indicated by the CCD. 

For organic acids, amino acids, and each compound whose ionization changes depending on pH, the chemical shift varies accordingly. These spectral variations may affect or invalidate the results of the multivariate analysis, whose basic principle is that the corresponding signals in different samples must have the same chemical shift. To minimize shifts, the pH of the samples with a buffered solution was standardized. To 9 mL of each mixture, 1 mL of 1 M KH_2_PO_4_ was added and the pH was measured; eventually, it was adjusted to 3.1–3.2 by using small amounts of 1 M HCl. An aliquot of 630 μL of this buffered mixture were then added to 70 μL of 13 mM 3-(trimethylsilyl)propionic-2,2,3,3-d_4_ acid sodium salt (TSP) and 0.1% sodium azide in 99.9% deuterium oxide (D_2_O). TSP was employed for internal referencing of ^1^H chemical shifts, while sodium azide as the preserving agent. The final solution (700 μL) was transferred into a WILMAD^®^ NMR tube, 5 mm, Ultra-Imperial grade, L 7 in., 528-PP purchased from Sigma-Aldrich (Milan, Italy). All chemicals and solvents were of analytical grade and they were purchased from Sigma-Aldrich.

### 3.2. NMR Spectroscopy

One-dimensional ^1^H NMR spectra of juice mixtures were acquired with a Bruker FT-NMR Avance III HD 600 MHz spectrometer (Ettlingen, Germany), and all the NMR experiments were performed at 300 °K. After 10 min of thermal equilibration inside the probe, the solvent (D_2_O) was locked to assure the maximum sensitivity, the probe was manually tuned and matched, and the 90° pulse was calibrated; subsequently, the power level for pre-saturation was calculated, and finally the receiver gain was automatically set. 1D NOESY experiments were acquired by using the Bruker sequence “noesygppr1d” in order to suppress the water signal. The pre-saturation frequency was adjusted for each sample before acquisition. The acquisition parameters were as follows: time domain (number of data points), 64 K; dummy scans, 0; number of scans, 32; acquisition time, 3.90 s; delay time, 8 s; spectral width, 14 ppm (8403.4 Hz), fid resolution, 0.2564 Hz; digitization mode, baseopt, total acquisition time, 6 min 22 s. 

### 3.3. Spectra Pretreatment

The application of the ^1^H NMR technique to juice samples generates complex and crowded spectra, which need to be previously pre-treated and then analyzed by chemometric tools to handle the high amount of information contained in the raw data. Assuming that peak intensities are directly proportional to the concentration of compounds, each spectral point was used as an absolute intensity value, without performing any type of signal integration. This approach allows for overcoming issues related to the understanding and integration of overlapped signals. First, ^1^H NMR spectra were phased and calibrated using the TSP signal for chemical shifts referencing, and the baseline was adjusted. All spectra processing was performed using TopSpin 3.5 software package (Bruker Biospin GmbH Rheinstetten). Each spectrum generates a 64 K data point file. In order to perform the spectra alignment, the files from each sample were exported and collected in a dataset consisting in 64 K spectral variables for 75 samples (60 calibration samples plus 15 test set samples). The alignment was performed by using Icoshift 1.0 toolbox for MATLAB^®^ (Mathworks Inc., Natick, MA, USA) to reduce the lack of homogeneity in chemical shifts that principally occurs for pH-dependent signals [18]. Afterwards, in order to reduce the number of data points and manipulate datasets more easily, non-significant spectral regions were deleted to obtain a new dataset with 34,740 data points and, finally, the resolution was reduced by selecting one out of every 10 points, thus obtaining 3474 data points for each spectrum.

### 3.4. Statistical Analysis

With a view to performing the statistical analysis, the dataset used for the alignment and resolution reduction (75 samples) was again split into two datasets, i.e., one containing the 60 samples for calibration and the second containing the 15 samples for the external validation test set. All multivariate data analyses and calculations were performed by using the software PLS_Toolbox 5.2.2 (Eigenvector Research Inc., Manson, WA, USA) for MATLAB^®^. The PLS method is based on the SIMPLS algorithm. All data were mean-centered and scaled by applying the Pareto scaling method, which is useful when spectral noise is expected to be proportional to the standard deviation square root of variables. Pareto scaling can reduce the relative importance of large values, keeping the data structure partially intact [19]. 

Partial least squares regression (PLS) is an extension of the multiple linear regression model that does not impose the restrictions employed by discriminant analysis, principal components regression, or canonical correlation. In PLS, prediction functions are represented by factors extracted from the Y’XX’Y matrix, and it is probably the least restrictive of the multiple linear regression extensions. PLS analysis was performed using a venetian-blind internal cross-validation method with the number of data splitting set to 7. The number of factors (latent variables) in the models and the model performance were assessed by using the root mean square error of calibration (RMSEC) and the root mean square error of cross-validation (REMSCV). The presence of potential spectral outliers was verified by applying a 95% confidence interval to the Q residuals and Hotelling’s T^2^ scores (Figure 3). 

The final regression model was assessed by the coefficients of determination for calibration, cross-validation and test sets (R^2^_CAL_, R^2^_CV_), the root mean square error of calibration and the root mean square error of cross-validation (RMSEC, RMSECV). The prediction ability of the model was evaluated by external validation set (test set), through the coefficient of determination for the test set (R^2^_PRED_) and the root mean square error of prediction (RMSEP).

## 4. Conclusions

The present work proposes the use of ^1^H NMR applied to the development and optimization of multivariate calibration models to determine the relative concentration of four juices in blends. The PLS model was established by using different chemometric indicators (RMSEC, RMSECV, RMSEP, R^2^_CAL_, R^2^_CV_ and R^2^_PRED_). The results demonstrate the feasibility and several advantages of using ^1^H NMR spectral data in combination with multivariate data analysis to build a model capable of detecting adulterations in fruit juice.

## Figures and Tables

**Table 1:** PLS model results of four fruit juice samples.

Model	Number of Factors: 5 Variance: Y = 88.62%; X = 94.51%			
	RMSEC ^a^	RMSECV ^b^	RMSEP ^c^	R^2 d^
Apple	6.869	8.732	2.324	R^2^_CAL_ = 0.912
				R^2^_CV_ = 0.899
				R^2^_PRED_ = 0.987
Orange	6.333	9.435	4.435	R^2^_CAL_ = 0.914
				R^2^_CV_ = 0.882
				R^2^_PRED_ = 0.950
Pineapple	8.634	12.631	5.438	R^2^_CAL_ = 0.885
				R^2^_CV_ = 0.821
				R^2^_PRED_ = 0.946
Pomegranate	7.182	10.511	7.092	R^2^_CAL_ = 0.950
				R^2^_CV_ = 0.860
				R^2^_PRED_ = 0.929

^a^ RMSEC: root mean square error of calibration; ^b^ RMSECV: root mean square error of cross-validation; ^c^ RMSEP: root mean square error of prediction. ^d^ R^2^: coefficient of determination for calibration (CAL), cross-validation (CV) and prediction (P).

**Table 2:** Percentage composition of juice samples.

Sample	% Apple	% Orange	% Pineapple	% Pomegranate
1	100	0	0	0
2	0	100	0	0
3	0	0	100	0
4	0	0	0	100
5	43.75	6.25	43.75	6.25
6	25	25	25	25
7	6.25	6.25	43.75	43.75
8	43.75	6.25	6.25	43.75
9	25	25	25	25
10	19.5	19.5	19.5	41.5
11	10	10	10	70
12	33.33	33.33	0	33.33
13	31.82	4.54	31.82	31.82
14	33.33	33.33	33.33	0
15	6.25	43.75	43.75	6.25
16	0	33.33	33.33	33.33
17	10	70	10	10
18	19.51	41.46	19.51	19.51
19	19.51	19.51	41.46	19.51
20	10	10	70	10
21	25	25	25	25
22	31.82	31.82	4.54	31.82
23	6.25	43.75	6.25	43.75
24	4.54	31.82	31.82	31.82
25	33.33	0	33.33	33.33
26	43.75	43.75	6.25	6.25
27	31.82	31.82	31.82	4.54
28	41.46	19.51	19.51	19.51
29	25	25	25	25
30	70	10	10	10

**Table 3:** Percentage composition of test set samples.

Sample	% Apple	% Orange	% Pineapple	% Pomegranate
TS1	20	15	30	35
TS2	44.44	22.22	22.22	11.11
TS3	40	13	7	40
TS4	18.92	13.51	33.78	33.78
TS5	20	60	15	5
TS6	30	30	25	15
TS7	12	60	19	9
TS8	35	10	30	25
TS9	21	25	45	9
TS10	10	27	34	29
7 *	6.25	6.25	43.75	43.75
9 *	25	25	25	25
13 *	31.82	4.54	31.82	31.82
22 *	31.82	31.82	4.54	31.82
28 *	41.46	19.51	19.51	19.51

* CCD samples randomly selected.
